# Personalized Glucose Management With AI: Pilot Study Using a Multiarmed Bandit Approach

**DOI:** 10.2196/70826

**Published:** 2026-03-19

**Authors:** Shinji Hotta, Mikko Kytö, Saila Koivusalo, Seppo Heinonen, Pekka Marttinen

**Affiliations:** 1 Fujitsu Limited, 4-1-1 Kamikodanaka, Nakahara-ku, Kawasaki, Kanagawa, 211-8588, Japan, 81 44 777 1111; 2 Department of Computer Science, Aalto University, Espoo, Uusimaa, Finland; 3 Development and Strategy Unit, Helsinki University Hospital, University of Helsinki, Helsinki, Uusimaa, Finland; 4 Department of Computer Science, University of Helsinki, Helsinki, Uusimaa, Finland; 5 Department of Obstetrics and Gynecology, Helsinki University Hospital, University of Helsinki, Helsinki, Uusimaa, Finland

**Keywords:** diabetes, mobile intervention, personalization, dietary and exercise recommendation, glucose management, multiarmed bandit

## Abstract

**Background:**

Personalized behavioral recommendations through mobile apps have proven effective in preventing serious chronic diseases such as diabetes. Recent studies have primarily focused on optimizing personalized recommendations using reinforcement learning. However, the main problem with these approaches is that they focus on behavioral changes and overlook clinical outcomes.

**Objective:**

This study aimed to propose a method for online planning of dietary and exercise recommendations to optimize postprandial glucose levels through behavioral changes directly.

**Methods:**

The proposed method is a multiarmed bandit based on a two-stage reward prediction model, where an action is a combination of the total carbohydrate intake and postprandial walking duration, and the reward is the reduction in postprandial glucose levels. We implemented the prediction of the reward for each action based on the predicted behavioral responses to an action, and subsequently, the postprandial glycemic response.

**Results:**

In a simulation experiment, we demonstrated that the proposed online algorithm can significantly improve postprandial glucose levels with personalized recommendations, compared to the randomized policy. Furthermore, we conducted a small real-world experiment with a simplified proposed method involving a single update of the recommendation policy into a personalized one. For 6 participants, compared to the randomized policy, we observed a 23% improvement, on average, in actual glucose responses along with the behavioral adherence to the recommendations concerning carbohydrate intake and postprandial walking.

**Conclusions:**

The preliminary effectiveness of the proposed method was demonstrated from both the simulation experiment and the small real-world experiment. However, further longitudinal real-world experiments in patients with diabetes are needed to validate and generalize the findings.

## Introduction

### Background

The global incidence of diabetes has increased considerably in recent years, accompanied by escalating disease severity. This progression has detrimental ramifications, including compromised quality of life, multifarious complications, and expensive surgical treatment. The medical expenses for diabetes are expected to increase to approximately US $2.5 trillion worldwide by 2030 [1]. This has facilitated the urgent need to reduce the severity of diabetes. The recent national clinical practice guidelines [2] state that the basic principle of this preventive treatment is to maintain glucose levels within the normal range. The central strategy to manage glucose levels is to maintain an appropriate lifestyle of eating and exercising [3], in addition to insulin or oral medication. In recent years, technological advances in continuous glucose monitoring devices have helped patients manage their glucose levels with mobile apps at any given time and place, thus promoting self-management [4,5]. However, it is difficult for patients to learn the optimal behavioral action to maintain glucose within a normal range [6]. Therefore, a personalized system that recommends individualized optimal behavioral actions is required to ensure that glucose levels do not deviate from the normal range in the future.

Over the past decade, it has become possible for patients with prediabetes to self-monitor their glucose levels, along with their lifestyle habits, such as diet and exercise, by visualizing them through an integrated mobile app [4,7]. While this has made it easier for patients to manage their own glucose levels daily, and maintaining high user adherence to such monitoring is achievable, as demonstrated by recent studies reporting over 90% daily adherence [8], learning the best behaviors to avoid abnormal glucose levels remains a significant hurdle [4]. For instance, the total grams of carbohydrates to be consumed or the total minutes of walking exercise to be performed vary from person to person. Therefore, the behavioral recommendations should be automatically generated for each patient by analyzing personal datasets collected daily.

### Literature Review

Thus far, there has been significant research on personalized behavioral recommendations through mobile apps for the prevention of chronic diseases, including diabetes [9], and a framework called just-in-time adaptive intervention has been established. In just-in-time adaptive intervention, to improve both the short-term and long-term outcomes, decision rules regarding intervention conditions, such as content, frequency, and timing of recommendations, are personally optimized [10]. For instance, in [11], to promote physical activity in patients with hypertension, many interventions with different conditions were allocated and performed on each patient’s timeline, and the best-fitting intervention conditions were identified based on the results of the interventional experiments. While this approach can robustly identify personally appropriate intervention conditions, its practical application for practitioners is constrained by the significant burden associated with prolonged experimental periods and the necessity for patients to undergo multiple interventions that may prove to be suboptimal. This underscores the need to develop more efficient and effective personalization methods.

With regard to the aforementioned factors, recent studies have focused on sequentially optimizing personal intervention conditions using reinforcement learning [12]. Yom-Tov et al [13] proposed a contextual bandit that optimized the message content of exercise interventions for patients with diabetes. In this study, an action is the message content, and a reward is the amount of activity after the recommendation. The individual optimization of message content can be adaptively performed by selecting an action based on a reward using Boltzmann sampling. Liao et al [14] proposed a framework that applies a multistep Markov decision process by introducing a user state, which represents how much intervention to increase daily walking has been received so far. This helps adjust both the intervention content and the intervention frequency to avoid user dropout caused by frequent interventions. However, while the focus of these existing methods is on patient behavior change, it is still inconclusive whether these methods can offer optimal interventions for improving clinical outcomes. For instance, in the case of diabetes, the extent to which the intervention lowers glucose levels is highly significant. In other words, further research is needed to identify the intervention conditions that can lower glucose levels most effectively to the target range.

Some studies have proposed optimizing clinical insulin interventions for people with more severe diabetes using reinforcement learning with an artificial pancreas to directly control their glucose levels [15,16]. When insulin is injected, the glucose level drops immediately. Therefore, the primary goal of such research is to determine the appropriate insulin dose and timing for each patient to ensure that the glucose level remains within the normal range. The action is the insulin dosage, the state is the user’s glucose level, and the reward is the normality of the glucose levels after insulin injection. The integration of reinforcement learning helped obtain the optimal insulin dosage for each patient [17]. However, in this type of study, the variations in action were limited to clinical and direct interventions, such as insulin injection, which are not directly controlled by the patient. Incorporating behavioral interventions, such as diet and exercise recommendations, in addition to clinical interventions, is currently regarded as future work [15,16].

### Objectives

Altogether, there is a lack of approaches that individually optimize behavioral recommendations such as diet and exercise to control glucose levels. In this paper, we propose a multiarmed bandit algorithm that can individually plan mobile behavioral interventions, making it easy for users to perform and reducing glucose levels after eating (ie, postprandial glucose, which is most likely to become abnormal in the daily lives of people with diabetes). The most novel aspect of the proposed method is the introduction of a two-stage reward prediction mechanism for each intervention: (1) prediction of the actual user behaviors that occur after the intervention, and (2) prediction of the postprandial glucose trajectory from these predicted behaviors.

**Figure 1.**
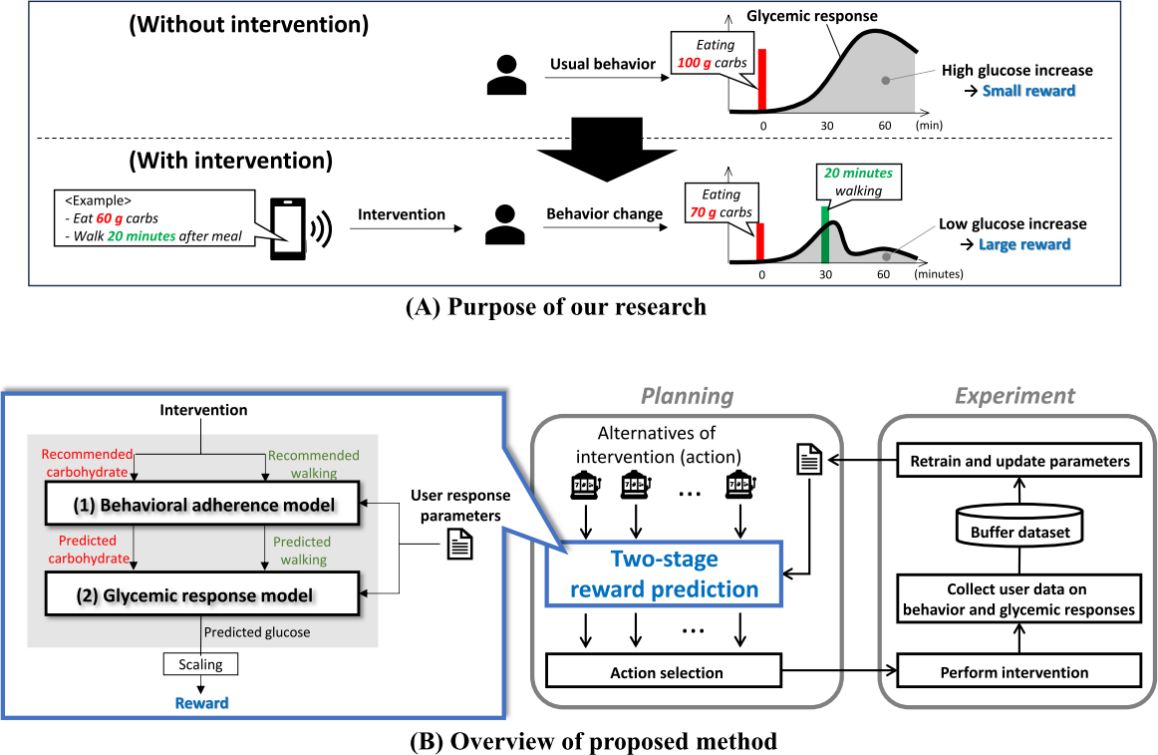
Purpose of our research (A) and overview of the proposed method (B).

An overview of the proposed method is provided in Figure 1. The aim is to lower postprandial glucose levels with the intervention (Figure 1A). Here, the action (or arm) is a mobile behavioral intervention that combines recommended amounts of carbohydrate intake and postprandial walking exercise, which have been proven to have specific effects on glucose levels [18,19]. Multiple alternative candidates for these recommended amounts are prepared in advance, and the optimal action for each user is selected based on the reward predicted in a two-stage manner, as provided in Figure 1B. In this reward prediction, the actual amounts of both carbohydrate intake and postprandial walking are predicted for each action candidate. For example, for carbohydrate intake, when a user receives a recommendation to take 60 g of carbohydrates, it is predicted that the participant will take 70 g because the recommended amount is small according to their dietary preference. Second, based on the predicted dietary and exercise behaviors, the temporal trajectory of postprandial glucose levels is predicted. Third, according to the predicted glucose trajectory, batched actions are selected using the ε-greedy method, based on the assumption that the greater the suppression of the increase in postprandial glucose, the higher the reward obtained by a user. Finally, after performing the selected actions using a real intervention, the behavioral adherence and glycemic response models are retrained using the buffer dataset of actual behaviors and glucose, and the parameters are updated accordingly. By repeating this experiential learning cycle, which is composed of action planning and real experiments, it is possible to fill the gap between each user’s real response and the virtually predicted response, which yields a more optimal action selection for lowering postprandial glucose levels. Furthermore, to accelerate this cycle, we suggest incorporating prior knowledge into the prediction models for retraining.

We demonstrate the effectiveness of the proposed method through simulation experiments and clarify the conditions under which the proposed method works best. Then, with a simplified algorithm based on these conditions, we conducted a small-scale mobile intervention experiment with healthy participants. The results provide preliminary evidence that the proposed method improves both behavior and glucose levels. To the best of our knowledge, this is the first attempt to (1) clarify how to optimize behavioral interventions for improving glucose levels and (2) demonstrate the effectiveness of the method through simulation experiments and real-world intervention experiments.

## Methods

### Overview

In the following subsections, we describe the problem setting, the details of the proposed method, the simulation experiment setup, and the real-world feasibility study setup. The proposed method is formulated as a model-based multiarmed bandit framework with a two-stage reward prediction model that integrates behavioral adherence modeling and glycemic response modeling to enable data-efficient personalization of dietary and exercise recommendations. Its effectiveness is evaluated through both simulation experiments and a real-world feasibility study.

### Problem Setting

The purpose of our method is to select and recommend the best action candidate for each user that is easy to perform and reduces postprandial glucose levels the most through interaction with the user. For this purpose, a multiarmed bandit algorithm is often used, as in previous literature [13,20,21]. The multiarmed bandit is one of the reinforcement learning methods. It continuously addresses the dilemma of selecting actions to gather more information (exploration) vs selecting actions to maximize immediate reward based on current knowledge (exploitation). Thus, a bandit algorithm can adjust this exploration-exploitation trade-off [22] by estimating the rewards associated with different actions.

In our method, the reward r can be defined as the function of the total increase in postprandial glucose levels after the target diet, which occurs after recommending an action A to a certain user. We mainly focus on postprandial glucose, given that glucose levels always rise after a meal and that deviations from the normal range of glucose levels occur most frequently after a meal [23,24].

**Figure 2.**
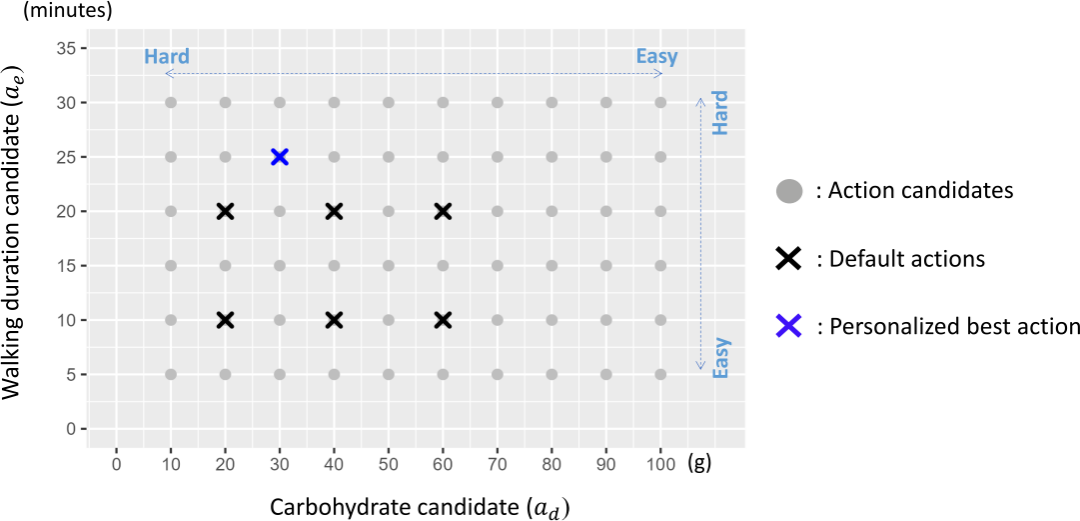
Illustration of action candidates. For default actions (×), the amount of carbohydrate intake is set within the range of 20‐60 g, and postprandial walking duration is set within the range of 10‐20 minutes. Although it is easy to adhere to taking a large amount of carbohydrate and walking for a few minutes, this leads to higher postprandial glucose levels. The main purpose is to find the best personalized action (×) that can achieve lower postprandial glucose levels while maintaining adherence for each user.

An action in our method A(∈A) can be represented as a combination of $A=\left\{a_{d},a_{e}\right\}$, where $a_{d}$ means a recommendation for “how much carbohydrates in grams a user should take at maximum in target diet,” and $a_{e}$ means a recommendation for “the minimum duration a user should walk just after target diet.” For clarity, users do not directly estimate carbohydrate content; instead, it is automatically calculated by a mobile app such as MyFitnessPal (MyFitnessPal, Inc) based on inputted food items. The accuracy of MyFitnessPal’s carbohydrate calculations has been rigorously validated, showing strong correlations (*r*=0.90) with established food composition databases [25]. Based on the assumption that each user has their own appropriate amounts for both actions, multiple action candidates are prepared in advance within a clinically plausible range. Regarding the amount of carbohydrate intake ($a_{d}$), we prepared 10 candidates in the range of 10‐100 g, referring to the low-carbohydrate diet recommendations [26]. In addition, regarding the amount of postprandial walking exercise ($a_{e}$), we prepared 6 candidates between 5 and 30 minutes based on the experimental results of the effect of walking exercise on glucose levels [19,27-29]. Therefore, there are 60 action candidates in the action set A based on previous research [19,26-29], as shown in Figure 2.

Here, there are 2 approaches for action selection in a multiarmed bandit, that is, the experience- and the model-based approaches. The former (eg, Upper Confidence Bound) evaluates each action based on the actual observed rewards, which enables robust evaluation but requires extensive exploration for all actions. In contrast, the latter (eg, Thompson Sampling) can reduce exploratory trials because it can estimate the reward (or value) for each action by learning the parameters of the reward distribution. Returning to our setup (Figure 1A), each observation of the reward (glucose level change) requires users to wear a glucose monitoring device, imposing a recurring physical burden. Therefore, it is important to reduce the burden of exploration trials on users for real applications, and we adopted this model-based approach to predict the reward for untried actions efficiently from limited observations.

Now, the reward prediction model is represented as follows:


r=R(A|Θ)

**Figure 3.**
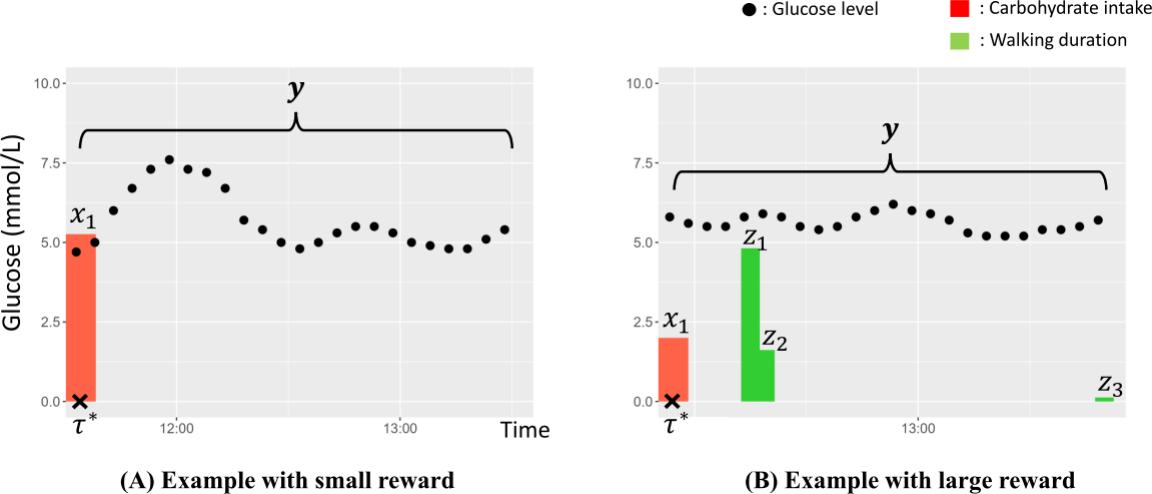
Two examples of observed data with a small reward (A) and a large reward (B). The greater the postprandial increase in glucose levels, the smaller the reward.

The input is an action A, and Θ is a model parameter set of a target user. Because Θ is unknown at the initial trial, it is sequentially updated when observing user responses to previously recommended actions. Here, the user response is composed of 3 variables, (x,y,z) as shown in Figure 3; x is the actual amount vector of each carbohydrate intake at the target diet, z is the actual walking duration vector of each walking event occurring after starting the target diet, and y is the time series of postprandial glucose levels after the start time of the target diet as $\tau^{*}$. Because the glucose level within 1 hour after a diet is of clinical importance, we focused on user responses within 90 minutes of starting the diet. As a result, for each action, the observed data are represented as D={A,x,y,z} and are added to the buffered dataset B after observation.

Here, glucose levels and behaviors in 1 observation “D” would involve high uncertainty because they may be affected by many factors. Thus, a parameter set “Θ” is updated in a Bayesian fashion when obtaining batched observation data containing “M” actions and “M” user responses. That is, as shown on the right side of Figure 1B, parameter learning is executed after recommending “M” actions to the users. This can be considered as a batched multiarmed bandits setting [30]. Hereafter, the method of selecting “M“ actions is referred to as the policy.

### Algorithm of the Proposed Method

In this subsection, we describe the proposed multiarmed bandit algorithm based on two-stage reward prediction. The pseudocode for the proposed algorithm is shown in Textbox 1. In this algorithm, the following processes are executed in the given order: (1) actions are recommended to users according to the predetermined policy, and batch observation data are obtained; (2) the parameters of the behavioral adherence and glycemic response models are retrained using these observation data; (3) the reward for each action candidate is predicted using a two-stage reward model with retrained parameters; and (4) finally, the next batch actions are selected based on the reward prediction result using the ε-greedy method.

These processes are repeated to improve the rewards and model parameters. This predictive model–based approach allows the algorithm to estimate potential outcomes of all possible actions based on learned models, rather than requiring direct, burdensome observation for every action candidate. In the first iteration, default actions are recommended for pure parameter exploration, aiming to gather diverse data for initial model parameter estimation. Afterward, as the number of iterations increases, more robust parameter learning can be performed with more observational data, resulting in accurate reward prediction and action selection. However, if the convergence of this parameter learning requires long-term (eg, several months of) observation data owing to the uncertainty contained in the observation, the user would drop out due to the burden of such time-consuming experiments for collecting data. To prevent this, we propose introducing prior knowledge for each parameter and performing Bayesian learning.

Textbox 1. **Algorithm 1: multiarmed bandit with two-stage reward prediction.**

**INPUT**: maximum iteration number *I*, batch size *M*, default policy $\pi_{1}$, and action set A.

1. // ***Repeat experimental learning cycle of intervention experiment and action planning for a user***
2. **For** *i* **in** 1:*I*
3. // *Perform intervention*
4.   **For** *j* **in** 1:*M*
5.    Recommend the action $A_{j}=\pi_{i}\;(j)$ for the user
6.    Observe the user responses $y_{i,j},x_{i,j},z_{i,j}$ …………………………………………………… (†)
7.   **End**
8.   Update buffered dataset $B_{i}$ by adding new batch data$D_{i}=\overset{M}{\underset{j=1}{⋃}}\left\{A_{i,j},y_{i,j},x_{i,j},z_{i,j}\right\}$
9.   // *Train each user model from subset of buffered dataset*
10.   Train the parameters $\theta_{d}(i)$ in dietary adherence model from the subset data$B_{i}^{\left(d\right)}$
11.   Train the parameters $\theta_{e}(i)$ in exercise adherence model from the subset data
12.   Train the parameters $\theta_{g}(i)$ in glycemic response model from the subset data
13.   // *Two-stage reward prediction for each action*
14.   **For** $A_{k}\in A$
15.    Predict dietary behavior $x_{k}^{*}$ with $\theta_{d}(i)$ under action $A_{k}$
16.    Predict exercise behavior $z_{k}^{*}$ with $\theta_{e}(i)$ under action $A_{k}$
17.    Predict glucose increase trajectory $y_{k}^{*}$ with $\theta_{g}(i)$ under predicted behaviors $x_{k}^{*},z_{k}^{*},$
18.    Obtain reward $r_{k}$ from glucose increase trajectory $y_{k}^{*}$
19.   **End**
20.   // *Action selection for next intervention*
21.   **If** *Rand*() < ε
22.    Select actions randomly as $\pi_{i+1}=\pi_{random}=\overset{M}{\underset{m=1}{⋃}}Random\left(A\right)$ // for exploration
23.   **Else**
24.    Select actions greedy as $\pi_{i+1}=\pi^{*}=\overset{M}{\underset{m=1}{⋃}}\left\{\underset{k}{argmax}\;r_{k}\right\}$ // for exploitation
25.   **End**
26. **End**

### Two-Stage Reward Prediction Model

Here, we first focus on describing a two-stage reward prediction model that integrates behavioral adherence models into a glycemic response model. The starting point for modeling is based on previous clinical findings, which state that the changes in postprandial glucose are highly dependent on the amount of carbohydrates an individual takes [31] and the amount of exercise performed after a meal [19,27-29]. Furthermore, these dietary and exercise behaviors are assumed to depend on the kind of actions recommended immediately beforehand. Based on these premises, the reward prediction model is described as follows:


(1) $$R\left(A|\Theta \right)=f\left(y^{*}\left(A|\Theta \right)\right)$$

(2) $$y^{*}\left(A|\Theta \right)=g\left(x^{*},z^{*}|\theta_{g}\right)$$

(3) $$x^{*}=h_{d}\left(a_{d}|\theta_{d}\right),\;\;\;z^{*}=h_{e}\left(a_{e}|\theta_{e}\right)$$

where R(A|Θ) is the reward, $y^{*}\left(A|\Theta \right)$ is the glucose time series, $x^{*}$ is the amount of carbohydrate intake, and $z^{*}$ is the walking duration. Further, $\Theta =\left\{\theta_{g},\theta_{d},\theta_{e}\right\}$ and $\theta_{g},\theta_{d},\theta_{e}$ represent parameters in a glycemic response model, a carbohydrate intake model, and a postprandial exercise model, respectively. These parameters are defined for each user. In actual reward prediction, the first stage is to predict the dietary and exercise behaviors using Equation 3 with behavior adherence models $h_{d}\left(\cdot \right)$ and $h_{e}\left(\cdot \right)$, and the second stage predicts the trajectory of the postprandial glucose increase $y^{*}$ using Equation 2 with the predicted behaviors ($x^{*},z^{*}$). Finally, the glucose increase trajectory is converted into a reward scale using Equation 1.

### Behavior Adherence Model

The first stage is behavior prediction. Because each user receives a specific action immediately beforehand, we can assume that they act according to this action. However, certain actions are difficult to execute in real-world scenarios. For example, most users would find it easy to keep their carbohydrate intake less than 100 g, but it is difficult to consume less than 10 g. In addition, the actual reaction to reducing carbohydrate intake to 50 g may vary for each user. This is consistent with the claim in the theory of planned behavior [32], which states that behavioral intentions may be influenced by “perceived behavioral control*.*” Inspired by this, in the behavior adherence model, we hypothesize that there is a perceptual critical point specific to each user for each action candidate, as indicated in Figure 2, and that the actual behavioral tendency changes depending on this critical point. Specifically, as shown in Figure S1 (left) in Multimedia Appendix 1, we assume that if the recommended amount of carbohydrate $a_{d}$ is higher than the critical point $X_{th}$, the user is likely to try to behave in accordance with the action. In contrast, if $a_{d}$ is lower than $X_{th}$, the user is likely to ignore the action and behave normally.

Based on the aforementioned assumptions, the behavior adherence model of carbohydrate intake is represented by the following linear model:


(4) $$h_{d}\left(a_{d}|\theta_{d}\right)=\left\{ \begin{array}{l} a_{d}+e_{x1}\;\;\;\;\;\;\;\;\;\;\;\;\;\;\;\;\;\;\;\;\;\;\;\;\;\;\;\;\;\;\;\;\;\;\;\;\;\;\;\;\;\;\;\;\left(if\;\;a_{d}\geq X_{th}\right)\\ a_{d}+X_{norm}\left(\frac{X_{th}-a_{d}}{X_{th}}\right)+e_{x2}\;\;\left(if\;\;a_{d}<X_{th}\right) \end{array}\right.$$

where $\theta_{d}=\left\{X_{th},X_{norm}\right\}$ and $X_{th}$ is the perceptual critical point for a target user, $X_{norm}$ is a parameter that represents the normal carbohydrate intake at the target diet. We also introduce Gaussian noises $e_{x1}∼N\left(0,\sigma_{x1}\right)$ and $e_{x2}∼N\left(0,\sigma_{x2}\right)$ to represent the uncertainty of carbohydrate intake from other factors. Here, different variance parameters ($\sigma_{x1},\sigma_{x2}$) are set because the level of behavioral uncertainty may depend on whether the critical point is exceeded.

Subsequently, the adherence model for postprandial exercise is based on the same concept, as described for carbohydrate intake above. As shown in Figure S1 (right) in Multimedia Appendix 1, if the recommended walking duration $a_{e}$ included in the action is shorter than the perceptual critical point of each user, it is expected to be adhered to. Otherwise, the recommended action is not adhered to, that is, the actual walking duration tends to be zero. Specifically, the exercise adherence model is represented using the following linear model:


(5) $$h_{e}\left(a_{e}|\theta_{e}\right)=\left\{ \begin{array}{l} a_{e}+e_{z1}\;\;\;\;\;\;\;\;\;\;\;\;\;\;\;\;\;\;\;\;\;\;\;\;\;\;\;\;\;\;\;\;\;\;\;\;\;\;\;\;\;\;\left(if\;\;a_{e}\leq Z_{th}\right)\\ a_{e}-Z_{th}\left(\frac{a_{e}-Z_{th}}{Z_{max}-Z_{th}}\right)+e_{z2}\;\;\left(if\;\;a_{e}>Z_{th}\right) \end{array}\right.$$

where $\theta_{e}=\left\{Z_{th},Z_{max}\right\}$ and $Z_{th}$ is the perceptual critical point and $Z_{max}$ is the parameter that represents the upper bound of walking duration in terms of the user’s physical strength. In this model, it was assumed that the walking time is zero at the upper bound. Further, we introduce Gaussian noises $e_{z1}∼N\left(0,\sigma_{z1}\right)$ and $e_{z2}∼N\left(0,\sigma_{z2}\right)$ to represent the uncertainty of exercise behavior from other factors.

Based on these behavior adherence models, each user’s dietary behavior parameters $\theta_{d}$ and exercise behavior parameters $\theta_{e}$ are inferred according to the buffered observation data $B_{i}$ accumulated for each user at an iterative time point i. Then, by deploying the inferred parameters $\theta_{d}\left(i\right),\theta_{e}\left(i\right)$ into each corresponding model $h_{d}\left(\cdot \right),h_{e}\left(\cdot \right)$, the behaviors $x^{*}\left(i\right)$, $z^{*}\left(i\right)$ in response to each action candidate can be predicted.

### Glycemic Response Model

Next, the postprandial glucose trajectory $y^{*}$ is predicted based on each behavior $x^{*},z^{*}$ predicted for action A. Our approach models this trajectory based on the clinical findings. First, it is well known that glucose levels increase with carbohydrate intake. For example, Ashrafi et al [33] showed that the actual postprandial glucose trajectory could be fitted by a response curve to carbohydrate intake. Second, it has been proven that exercises, such as walking, immediately lower glucose levels, as previously mentioned in this “two-stage reward prediction model” subheading [19,27-29]. The mechanism is that energy expenditure in muscle tissues due to exercise causes glucose absorption for energy supply [34]. For example, Jankovic et al [35] and Xie and Wang [36] adopted a linear additive model to predict the glucose trajectory and showed that predictive performance was improved by adding the exercise effect on glucose as a factor, in addition to the carbohydrate intake effect. Based on this glucose modeling, we hypothesized that the observed postprandial glucose trajectory y can be represented based on the summation of the response curve $R_{d}$ to carbohydrate intake and the response curve $R_{e}$ to exercise to the baseline preprandial glucose level $y_{base}$, as follows [37]:


(6) $$y=y_{base}+R_{d}\left(x,\tau_{x}\right)+R_{e}\left(z,\tau_{z}\right)+e_{y}$$

Because the time range of the glucose trajectory that we focus on here is short-term, we assume that the baseline value $y_{base}$ is constant and substitute the average of the glucose history from 15 minutes before starting the target diet. Here, $e_{y}$ is the vector of Gaussian noise following $N\left(0,\sigma_{y}\right)$. The response curves $R_{d},R_{e}$ are represented by the following equations:


(7) $$R_{d}\left(x,\tau_{x}\right)=\sum_{i=1}^{P}h_{d_{i}}exp\left(\frac{-0.5{\left(\Delta_{i}-3\alpha_{d}\right)}^{2}}{\alpha_{d}^{2}}\right),\;h_{d_{i}}=\beta_{d}x_{i},\;\Delta_{i}=t-\tau_{x,i}$$

(8) $$R_{e}\left(z,\tau_{z}\right)=\sum_{j=1}^{Q}h_{e_{j}}exp\left(\frac{-0.5{\left(\Delta_{j}-3\alpha_{e}\right)}^{2}}{\alpha_{e}^{2}}\right),\;h_{e_{j}}=\beta_{e}z_{j},\;\Delta_{j}=t-\tau_{z,j}$$

where $\tau_{x,i},\tau_{z,j}$ represents the time of event occurrence of the *i*th carbohydrate intake or the *j*th walking event, respectively. Further, P represents the number of carbohydrates consumed within the target diet, usually P=1. Also, Q represents the number of walking events that occurred within 90 minutes of starting the target diet. In these equations, we adopted a bell-shaped function as the response curve, similar to that in previous works [33,38], owing to its interpretability and the small number of parameters. Some specific examples of each response curve, $R_{d},R_{e}$ are shown in Figure S2 in Multimedia Appendix 1. This response curve starts at the timing of event occurrence $\tau_{x,i},\tau_{z,j}$. The response curve is then amplified based on the amount of carbohydrate intake $x_{i}$ or the duration of each walk $z_{j}$. Here $\beta_{d},\beta_{e}$ is a parameter that represents the degree of amplification with respect to the volume of each behavior. Further, $\alpha_{d},\alpha_{e}$ is a parameter that represents the degree of time delay of the response. A hyperparameter of the prior is introduced for each parameter $\beta_{d},\alpha_{d},\beta_{e},\alpha_{e}$, and we describe how this can be obtained in the subsequent *“*Parameter Learning*”* subheading*.*

Based on the above setting, specifically defined by Equations 6-8, it can be seen that the parameters of our glycemic response model $\theta_{g}=\left\{\beta_{d},\alpha_{d},\beta_{e},\alpha_{e}\right\}$ for each user are estimated from the observed behavior sequence (x,z) and glucose trajectory (y), at each iteration point (i). In this parameter learning, we use the observed behaviors (x,z) rather than the predicted behaviors ($x^{*},z^{*}$) as training data to identify the parameters more accurately, reflecting the real relationship. In contrast, the predicted behaviors ($x^{*},z^{*}$) are used as inputs for reward prediction for the action candidate A. Accordingly, the predicted postprandial trajectory is represented as follows:


(9) $$g\left(x^{*},z^{*}|\theta_{g}\right)=R_{d}\left(x^{*},\tau_{x^{*}}|\beta_{d},\alpha_{d}\right)+R_{e}\left(z^{*},\tau_{z^{*}}|\beta_{e},\alpha_{e}\right)$$

where the baseline term is removed compared to Equation 6. This is because the baseline term does not contribute to the glucose increase and, hence, the reward. Then, $y^{*}\left(A|\Theta \right)=g\left(x^{*},z^{*}|\theta_{g}\right)$ represents the series of pure increase of postprandial glucose affected by behaviors. It should be noted that for each iteration (i), the estimated parameter values $\theta_{g}\left(i\right)$ are used as $\theta_{g}$ in these equations.

Finally, the glucose increase trajectory, $y^{*}$, is converted into a reward scale. The reward should be designed such that the smaller the degree of glucose increase, the higher the reward. We use the incremental area under the curve (iAUC), a traditional glucose metric [39], as the degree of glucose increase. Based on these factors, we perform reward conversion using the sigmoid function, as shown below:


(10) $$f\left(y^{*}\right)=\frac{1}{1+exp\left\{a\left(iAUC-b\right)\right\}}$$

$$iAUC=\sum_{t=\tau^{*}}^{\tau^{*}+T}y_{t}^{*}$$

where iAUC is the total value of the trajectory of glucose increase. As described in the *“*Problem Setting*”* subheading, $\tau^{*}$ denotes the start time of the diet, and T=90 minutes. a,b are parameters for the conversion from the iAUC to the reward scale. We set a=−3 and b=1 empirically, based on the distribution of the iAUC. Consequently, the reward value ranges between 0 and 1.

Thus, for each action candidate A, a reward calculated from postprandial glucose increase $R\left(A|\Theta \right)=f\left(y^{*}\left(A|\Theta \right)\right)$ is predicted based on our original two-stage prediction.

### Action Selection

After the reward is predicted for each action candidate, the policy for selecting batched actions for the real intervention is selected. Specifically, the action that maximizes the predicted reward is selected. However, the predicted reward is not always accurate due to overfitting caused by scarce training data at the initial stage. Moreover, if the action is always selected to maximize that reward, the real action for the user becomes fixed, and the observed user response subsequently exhibits a small variation, which also leads to overfitting. Therefore, we adopt the ε-greedy method for balancing exploration and exploitation. For exploitation, assuming that the prediction model is accurate, actions that maximize the reward (π) should be selected. For exploration, assuming that the prediction model needs to be trained more, actions are selected randomly ($\pi_{random}$). Such policies $\pi^{*}$ and $\pi_{random}$ are taken with probabilities of 1−ε and ε, respectively. Because parameter learning is performed in a batched manner, M actions are selected simultaneously for each policy. Therefore, $\pi^{*}$ and $\pi_{random}$ are represented as follows:


$$\pi^{*}=\overset{M}{\underset{m=1}{⋃}}\left\{\underset{k}{argmax}\;R\left(A_{k}|\Theta \right)\right\},\pi_{random}=\overset{M}{\underset{m=1}{⋃}}Random\left(A\right)$$

where, *Random*(A) represents an operation for extracting an element randomly from a set A. Specifically, in $\pi_{random}$, this operation is repeated M times independently.

### Parameter Learning

The learning of the parameter set $\Theta =\left\{\theta_{g},\theta_{d},\theta_{e}\right\}$ in the reward model is performed at each iteration time just after M-intervention experiments are finished, and the batched observation data are accumulated in the buffered dataset. In this learning, all the buffered datasets accumulated up to the ith iteration point are used. If the ith batch of data $D_{i}$ are observed, the training dataset at that time becomes $B_{i}=\overset{i}{\underset{j=1}{⋃}}D_{j}$. The batched data includes a history of 4 types of data (action, postprandial glucose level, amount of carbohydrate intake, and postprandial walking duration) for M target meals. This is represented as $D_{j}=\overset{M}{\underset{m=1}{⋃}}\left\{A_{j,m},y_{j,m},x_{j,m},z_{j,m}\right\}$. In the observation data, carbohydrate intake and postprandial walking can occur multiple times per target diet. Therefore, they are represented as vectors (recall Figure 3B). In practice, $\theta_{g},\theta_{d},\theta_{e}$ are separately trained from the following subsets of the buffer data $B_{i}^{\left(g\right)},B_{i}^{\left(d\right)},B_{i}^{\left(e\right)}$ for each parameter.


$$B_{i}^{\left(g\right)}=\overset{i}{\underset{j=1}{⋃}}\left[\overset{M}{\underset{m=1}{⋃}}\left\{y_{j,m},x_{j,m},z_{j,m}\right\}\right],$$

$$B_{i}^{\left(d\right)}=\overset{i}{\underset{j=1}{⋃}}\left[\overset{M}{\underset{m=1}{⋃}}\left\{A_{j,m},x_{j,m}\right\}\right],$$

$$B_{i}^{\left(e\right)}=\overset{i}{\underset{j=1}{⋃}}\left[\overset{M}{\underset{m=1}{⋃}}\left\{A_{j,m},z_{j,m}\right\}\right].$$

Here, $B_{i}^{\left(g\right)},B_{i}^{\left(d\right)},B_{i}^{\left(e\right)}$ are the training datasets for the glycemic response, dietary adherence, and exercise adherence models, respectively.

To improve the efficiency of this learning process, we introduce prior distributions for each parameter. Specifically, for obtaining prior information for the behavioral parameter sets $\theta_{d},\theta_{e}$, we ask each user to answer questions corresponding to each parameter beforehand and use the responses to define the prior distribution. For example, the question for the critical point $X_{th}$ is “If you were to reduce carbohydrates in your dinner, what would you say is the minimum amount of carbohydrates?” Then, the user’s answer is set to the mean $\mu_{X_{th}}$ of the Gaussian prior distribution $N\left(\mu_{X_{th}},\sigma_{X_{th}}\right)$. For the other parameters $X_{norm},Z_{th},Z_{max}$, each prior distribution is set similarly.

On the other hand, the glycemic parameter set $\theta_{g}$ cannot be clarified through user interviews because each parameter is related to a complex individual biological response. Therefore, we adopt a data-driven approach wherein the prior distribution is pretrained through the original upstream task. Before starting the first intervention experiment, we recruited additional participants belonging to the same disease group as the target users. Second, we collect a glucose dataset, $D^{\left(g\right)}=\overset{L}{\underset{l=1}{⋃}}\left\{y_{l},x_{l},z_{l}\right\}$ for L diet events from each participant. Third, this dataset is used to infer glycemic parameters $\theta_{g}$ for each participant. Finally, the distribution of these parameters $\theta_{g}$ among all participants is used as the Gaussian prior distribution of $\theta_{g}$. Specifically, the mean ${\tilde{\mu }}_{g}$ and the variance ${\tilde{\sigma }}_{g}$ of these parameters $\theta_{g}$ are set as $N\left({\tilde{\mu }}_{g},{\tilde{\sigma }}_{g}\right)$. Here, each parameter $\theta_{g}$ is assumed to be generated independently.

Through this process, each prior distribution of the parameter set $\theta_{g},\theta_{d},\theta_{e}$ is obtained before the first intervention experiment, and this prior is used for Bayesian parameter learning at every iteration time point. This parameter learning is performed by executing a Markov Chain Monte Carlo simulation with the No U-Turn Sampler implemented in RStan (Stan Development Team) [40].

### Simulation Experiment Setup

We studied in a simulation experiment whether our online algorithm can improve postprandial glucose levels with personalized recommendations. In addition, to carry out real experiments with real personalized recommendations, we investigated the best setting for the proposed method to maximize performance. Specifically, we aim to clarify the following questions through simulation experiments:

- Q1: In action selection, how should the trade-off point between exploration and exploitation be set to maximize the cumulative reward?
- Q2: How fast does parameter learning converge when introducing a prior distribution into the reward prediction model?

To answer these questions, we set up several virtual users who exhibited different behavioral and glycemic responses, performed repeated action planning and pseudo-intervention experiments using the proposed method for each user, and evaluated these experimental results to address the above questions. At the same time, we also show that by introducing the proposed method, the reward performance can be improved compared to selecting default actions uniformly.

### Virtual User Setting

In the real world, behavioral tendencies and glycemic responses differ from user to user. Therefore, in this simulation, a parameter set $\theta_{g},\theta_{d},\theta_{e}$ for each user was set to ensure that virtual users exhibit response patterns as diverse as possible. Table 1 shows the actual settings of parameter values for each user. The total number of virtual users was 10.

**Table 1.** User settings for each parameter.

Virtual user	Dietary adherence model		Exercise adherence model		Glycemic response model			
	$X_{th}$(g)^a^	$X_{norm}$(g)^b^	$Z_{th}$(min)^a^	$Z_{max}$(min)^c^	$\beta_{d}$ ^d^	$\alpha_{d}$ ^e^	$\beta_{e}$ ^d^	$\alpha_{e}$ ^e^
ID1	50	100	20	60	0.05	20	−0.15	6.0
ID2	30	60	10	40	0.05	20	−0.20	8.0
ID3	70	120	30	80	0.05	20	−0.15	6.0
ID4	90	120	5	40	0.05	20	−0.20	8.0
ID5	50	100	20	60	0.04	15	−0.15	6.0
ID6	70	120	10	60	0.04	15	−0.20	8.0
ID7	30	60	30	80	0.04	15	−0.15	6.0
ID8	90	120	5	30	0.04	15	−0.20	8.0
ID9	10	40	30	80	0.04	15	−0.20	6.0
ID10	10	40	5	30	0.05	20	−0.20	8.0

a X_th_ and Z_th_: perceptual critical points for carbohydrate intake and postprandial walking duration (Equations 4 and 5).

b X_norm_: normal carbohydrate intake (Equation 4).

c Z_max_: upper bound of walking duration (Equation 5).

d β_d_ and β_e_: the degree of glycemic amplification of carbohydrate intake and postprandial walking duration (Equations 7 and 8).

e α_d_ and α_e_: the degree of time delay of the glycemic response to carbohydrate intake and postprandial walking duration (Equations 7 and 8).

As an example of user diversity, it is difficult for user ID4 to reduce carbohydrate intake because of $X_{th}=90$, whereas user ID9, with $X_{th}=10$ finds it easy to reduce carbohydrate intake. User ID4 also has $\beta_{d}=0.05$, indicating a greater glycemic response to carbohydrate intake, while user ID5 has a lower glycemic response due to $\beta_{d}=0.04$. Thus, each parameter value was set differently for different users. The value ranges of the behavior parameters $\theta_{d},\theta_{e}$ were within the range of the action set shown in Figure 2. These ranges were defined following established dietary and exercise guidelines. Specifically, the minimum carbohydrate intake ($X_{th}$) for virtual users was set with reference to “very low-carbohydrate” diets (20‐50 g/d) and “low-carbohydrate” diets (<130 g/d) as defined by Oh et al [41], while the normal carbohydrate intake ($X_{norm}$) was guided by typical daily carbohydrate intake levels (200‐325 g/d) as described in the literature [26]. For the parameters of postprandial walking durations ($Z_{th},Z_{max}$), typical ranges in prior experimental studies (5‐30 minutes) [19,27,29] and recommendations for at least 10 minutes of walking [27] informed the setting of $Z_{th}$, and cases of walking up to 90 minutes [28] guided $Z_{max}$. To introduce a diverse range of realistic individual differences among virtual users, slight deviations from these foundational literature values were sometimes permitted. Nevertheless, the chosen parameter ranges for virtual users in Table 1 do not drastically diverge from the prior information collected from real participants (in Table 2), suggesting that the simulated settings are not unrealistic. In addition, the value ranges of the glycemic parameters $\theta_{g}$ were set based on the prior knowledge obtained in the upstream task.

**Table 2.** Prior information on behavioral parameters.

Parameter	Questionnaire	ID1	ID2	ID3	ID4	ID5	ID6
$X_{th}$	“If you were to reduce carbohydrates in your target diet, what would you say is the minimum amount of carbohydrates?”	50 g	50 g	30 g	30 g	50 g	50 g
$X_{norm}$	“How much carbohydrates in grams do you typically consume in your target diet?”	110 g	90 g	50 g	50 g	70 g	150 g
$Z_{th}$	“If you were to walk after your target meal, how long do you think you could walk each day?”	30 minutes	15 minutes	15 minutes	20 minutes	25 minutes	5 minutes
$Z_{max}$	“Based on your physical fitness, how long do you think you can continue walking?”	60 minutes	60 minutes	180 minutes	60 minutes	40 minutes	120 minutes

#### User Response Simulator

Next, to perform the simulation experiment, it is necessary to generate a pseudo-response to any action according to each user setting. Therefore, we developed a user-response simulator that outputs 3 responses (dietary, exercise, and glycemic) to 2 inputs (an action and each parameter value), as shown in Figure S3 in Multimedia Appendix 1.

In this simulator, the simulated dietary behavior and exercise behavior for an action are the first outputs, and a simulated glucose trajectory is generated based on these behaviors. First, the simulated behaviors are pre-estimated according to the corresponding behavior adherence model in Eqs 4 and 5 by setting the input parameter values of the virtual user and input action $A=\left\{a_{d},a_{e}\right\}$. Subsequently, by perturbing the simulated behaviors by adding Gaussian noise, we obtain the final simulated behaviors $\overset{˘}{x},\overset{˘}{z}$. Here, the perturbation noise for carbohydrate intake followed N(0,10), and the noise for postprandial walking duration followed N(0,5). Subsequently, these simulated behaviors, $\overset{˘}{x},\overset{˘}{z}$ are input into the glycemic response model (Equation 9), and the obtained trajectory is then perturbed by adding Gaussian noise to generate the final simulated glucose trajectory $\overset{˘}{y}$. In this perturbation, a time-independent noise following N(0,0.02) was added. Figure S4 in Multimedia Appendix 1 shows a representative example of these simulated responses.

By substituting the part (†) of the pseudocode in Algorithm 1 (Textbox 1) with the above simulator, it is possible to simulate repeated experiential learning cycles of action planning and intervention experiments, as shown in Figure 1B, for each virtual user.

#### Simulation Evaluation Settings

To answer the question (Q1), we tracked how the actual cumulative reward changes depending on the settings of the proposed method while changing them. One of the most significant settings is the balance of exploration and exploitation in action selection, which is determined according to the set value of ε, a hyperparameter of the proposed method. Therefore, we set multiple patterns of ε values and investigated which setting yielded the highest cumulative reward. For these value patterns, we considered ε=1.0 for random selection, ε=0.5 for high, ε=0.2 for medium, and ε=0.01 for low condition of ε.

Another significant setting is the introduction of prior distributions for each model parameter. Thus, we also investigated how the cumulative reward varied depending on whether the prior distribution was used in parameter learning. In this simulation, prior knowledge of the behavioral parameters $\left\{X_{th},X_{norm},Z_{th},Z_{max}\right\}$ was pseudo-generated by adding Gaussian noise to the true parameter values. This may be attributed to the fact that the results of the survey questionnaire are subject to the human cognitive errors of each user regarding their true behavior, and these cognitive errors are assumed to follow Gaussian noise. On the other hand, to set the prior distribution of the glycemic parameters $\left\{\beta_{d},\alpha_{d},\beta_{e},\alpha_{e}\right\}$, the mean and the variance of the true values among all users were substituted. The initial batch of 6 actions was based on past clinical findings (refer to Figure 2). From the second iteration (i≥2), action selection was performed by integrating the proposed method under each setting.

### Metrics

Specifically, the cumulative reward of the virtual user (n) at iteration of the experiential learning cycle (i) can be represented as follows:


$$G_{n}\left(i\right)=\frac{1}{i\times D}\sum_{j=1}^{i}\sum_{d=1}^{D}r_{j,d,n}$$

where $r_{j,d,n}$ represents the actual reward transformed by Equation 10 from the glucose trajectory $\overset{˘}{y}$ of the virtual user n after the dth target diet in the jth intervention experiment. Further, D is the number of target diets included in each experiment; and D=6 was used in the simulation. The average cumulative reward of N virtual users, which is used as an evaluation metric, can be represented as follows:


$$J\left(i\right)=\frac{1}{N}\sum_{n=1}^{N}G_{n}\left(i\right)$$

By increasing i to the maximum iteration time I, we can track how the metric J(i) changes depending on each condition of ε. Considering the burden on users in real experiments, I was set to 10. This means that each user must continue wearing the necessary sensors to collect user responses for a maximum of 60 meals when D=6.

In this simulation, the true value of each parameter for each user was known, as listed in Table 1. This enables the investigation of the estimation error of each parameter for any setting of our method. To answer the question (Q2), we investigated how the estimation error changes as the iteration time increases (ie, the amount of training data), with and without prior distribution. The estimation error was defined as follows:


$$\epsilon_{p}(i)=\frac{\left|{\hat{\theta }}_{p}(i)-\theta_{p}\right|}{|\theta_{p}|}\times 100$$

This corresponds to the standard error (%). p is the parameter number, $\theta_{p}$ is the true value of the parameter in Table 1, and ${\hat{\theta }}_{p}\left(i\right)$ is the estimated value of the parameter in the ith iteration time.

### Real-World Feasibility Study Setup

Additionally, we conducted a preliminary set of short intervention experiments with real users using the proposed method, based on the simulation results. This experiment was positioned as a pilot study before conducting a full-scale study. The purpose of this experiment was to explore the feasibility of whether postprandial glucose levels can be improved in real users after recommending actions updated by the proposed method compared to default actions. Accordingly, in this study, we compared the performance when default actions were selected in the first experiment (referred to as the “default policy”) with the performance when actions were selected by the proposed method in the second experiment (referred to as the “optimized policy”) using a within-participant study design, to evaluate the feasibility of the proposed method. The optimized policy was updated from the observed participant responses in the first experiment, following the proposed method.

### Experimental Protocol

First, we recruited 6 healthy participants (4 men and 2 women) as real users from April 2023 to July 2023. Because this experiment was the first feasibility study, the target group was healthy rather than diabetic. All participants were researchers at Aalto University and the University of Helsinki.

Next, each participant was assigned the goal of reducing postprandial glucose levels to prevent diabetes. To achieve this goal, the participants received daily recommendations for the target diet, including total carbohydrate intake and postprandial walking duration, via a dedicated smartphone app. The target diet was either dinner or lunch each day, and the participants chose according to their lifestyle preferences. While the recommendations in the first experiment were preset to default actions, those in the subsequent experiment were preset to actions selected by the proposed method. Participants were free to choose whether to follow the recommendations and to act accordingly. To collect a participant’s behavioral and glycemic responses to the recommendation actions, they wore a continuous glucose monitoring device and an activity tracker.

This process of mobile intervention and data collection for each participant was carried out for 6 days in the first experiment and for 3 days in the second experiment. Because the first experiment corresponded primarily to the exploration phase and the second experiment corresponded to the exploitation phase, the number of days in the second experiment was set to be short.

### Recommendation of Action

An action was delivered as a recommendation in a notification app on a dedicated smartphone (iPhone 12), which was provided to each participant. In the notification app, a dedicated message including the action was delivered along with a notification sound, a few hours before the target diet. The frequency and duration of this recommendation were predetermined according to participants’ preferences.

An example of this message appearing on the smartphone screen is “Eat less than 60 g of carbohydrates at dinner, and walk for more than 20 minutes just after dinner.” Depending on the selected action, the amount of carbohydrate and postprandial walking duration in the message changed. For default actions in the first experiment, carbohydrate intake ($a_{d}$) was within the range of 20‐80 g, and the postprandial walking duration ($a_{e}$) was within the range of 5‐20 minutes, considering the participants’ preferences and previous literature [19,26-29]. For example, in the second experiment, actions were selected based on the two-stage reward prediction results of each action candidate; therefore, they were not always within this range. This reward prediction was attained by setting the parameters learned from the user response data obtained in the first experiment.

### Response Data Collection

**Figure 4.**
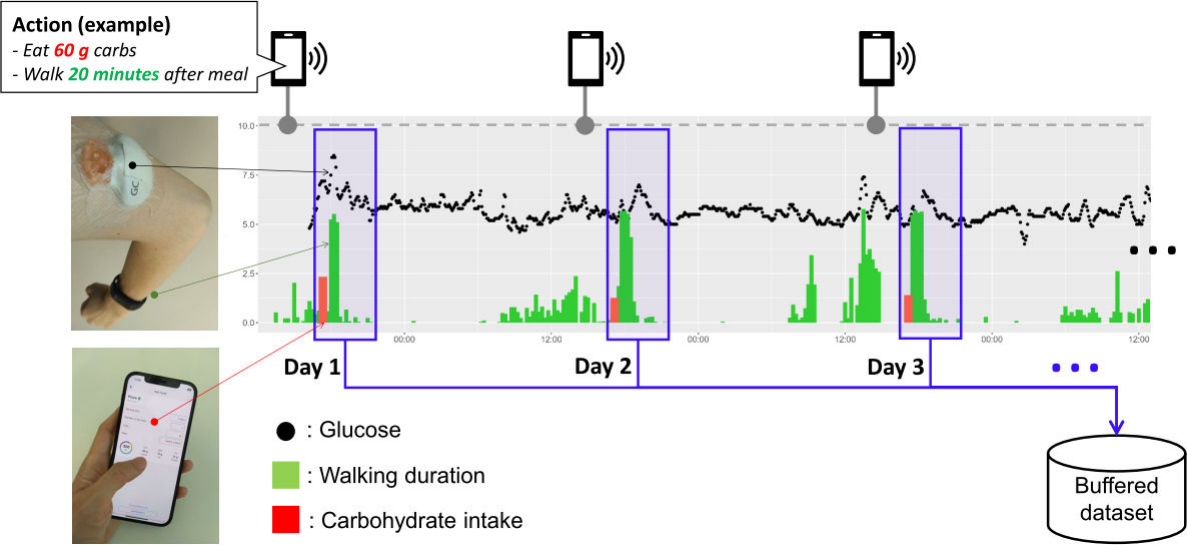
Illustration of response data collection.

For user response data, we collected data related to the participant’s continuous glucose levels, physical activity, and food records. Figure 4 illustrates how the data were collected. Continuous glucose measurements were collected every 5 minutes using a Guardian Connect medical device (Medtronic Inc), which was inserted on the participant’s skin. At the same time, physical activity data were collected using the activity tracker Vivosmart 3 (Garmin Ltd), which was worn on the participant’s arm. To mitigate erroneous detection of walking from various daily movements, the device identifies a walking event when characteristic arm movements indicative of continuous walking are detected for a duration exceeding a certain minimum threshold. The physical activity data included the start time and duration of each walking event that was automatically detected by the device. A dedicated fitness tracker was chosen over participants’ personal smartphones to ensure data consistency and reliability across participants, as smartphone usage and carrying patterns (eg, in a pocket or bag) can vary significantly and lead to inconsistent activity detection. This approach also minimizes issues related to battery drain on personal devices and addresses potential privacy concerns by avoiding continuous access to personal smartphone data. The glucose and activity data were continuously sent to a dedicated iPhone through a Bluetooth connection with each device. In addition, a food diary, including the amount of carbohydrates and the start time of each target diet, was recorded manually by the participant. The carbohydrate content was automatically calculated using the mobile app MyFitnessPal by inputting all food contents and amounts.

Finally, because we focus on the improvement of postprandial glucose levels, we extracted postprandial segment data from the longitudinal time series as user response data for each action. Specifically, the segment data were defined from the start of each target diet until 90 minutes later, as shown by the blue frames in Figure 4. Each data segment was added to a buffered dataset.

### Metrics

The evaluation was based on whether the reward related to postprandial glucose increased in the second experiment compared to the first. Therefore, we set the following metric as the actual reward average $J^{\prime }\left(i\right)$ for all diets of all *N* participants in the *i*th experiment, and this metric was compared between the first and second experiments.


$$J^{\prime }\left(i\right)=\frac{1}{N}\sum_{n=1}^{N}G_{n}^{\prime }\left(i\right),G_{n}^{\prime }\left(i\right)=\frac{1}{D}\sum_{d=1}^{D}r_{i,d,n}$$

It should be noted that the actual reward $r_{i,d,k}$ is transformed by Equation 10 from the glucose increase trajectory, which is obtained by subtracting its baseline derived from preprandial glucose levels from the observed glucose trajectory y.

### Acquisition of Prior Knowledge

To introduce the prior distribution of the behavioral parameters, each participant was asked to answer a questionnaire before starting the experiment. As described in the “Parameter Learning” subheading, the result was set as the mean value of the prior distribution of the corresponding parameter. Table 2 shows the actual answers for each parameter together with the corresponding question. It can be observed that the values vary according to the participant. As for glycemic parameters, we estimated the prior distribution $N\left({\tilde{\mu }}_{g},{\tilde{\sigma }}_{g}\right)$, which is common to target participants, using the glucose dataset from other participants. To obtain this, we recruited 4 additional healthy participants and collected continuous glucose levels, physical activity, and food records around lunchtime over 6 days for each participant in the same manner, as described previously. Consequently, a total of 6 segment data were obtained for each participant, as shown in Figure 4. Then, after estimating the glycemic parameters $\theta_{g}$ for each participant using his segment data, the parameters ${\tilde{\mu }}_{g},{\tilde{\sigma }}_{g}$ were calculated among 4 participants, as described in the “Parameter Learning” subheading in this section. As a result of this calculation, we obtained ${\tilde{\mu }}_{g}=\left\{{\tilde{\mu }}_{\beta_{d}},{\tilde{\mu }}_{\beta_{e}},{\tilde{\mu }}_{\alpha_{d}},{\tilde{\mu }}_{\alpha_{e}}\right\}=\left\{0.047,-0.148,19.07,6.22\right\}$ and ${\tilde{\sigma }}_{g}=\left\{{\tilde{\sigma }}_{\beta_{d}},{\tilde{\sigma }}_{\beta_{e}},{\tilde{\sigma }}_{\alpha_{d}},{\tilde{\sigma }}_{\alpha_{e}}\right\}=\left\{0.028,0.035,1.91,0.39\right\}$.

### Ethical Considerations

The study was approved by the Aalto University Research Ethics Committee (Treatment Planning Project/08.06/2023). Written informed consent was obtained from all participants before the start of the experiment. Direct identifiers were removed, and data were pseudonymized and stored securely. No personal data were shared with third parties. Additionally, to ensure participant safety, participants were informed orally and via the consent form that they could withdraw at any time if they experienced discomfort or unease.

## Results

### Simulation Experiment Results

**Figure 5.**
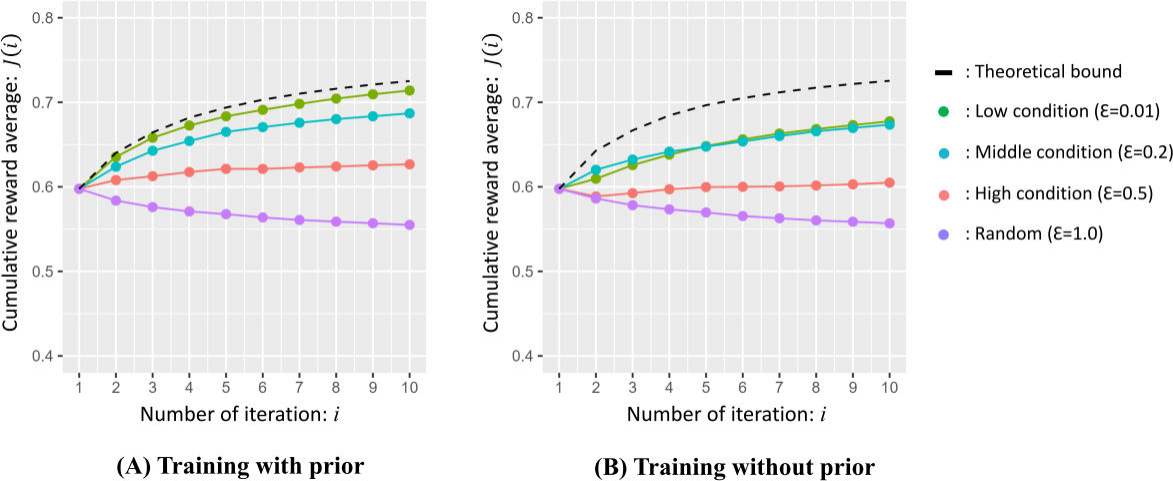
Simulation results of average cumulative reward with prior distribution “(A)” and without prior distribution “(B)” of the proposed method.

We present the simulated results of the average cumulative rewards (J(i)) with and without prior distribution in Figure 5. First, in any case, when an action selection by the proposed method is adopted (ie, when ε is other than 1.0), we can confirm that the cumulative reward gradually increases from the second iteration (i=2) after executing default actions at the first iteration (i=1). This demonstrates the effectiveness of action selection by the proposed method in the second and subsequent iterations (i≥2). Regarding question Q1, by focusing on the variation of ε, we can also confirm that the smaller the value of ε, the greater the accumulated reward, and the maximum cumulative reward is obtained when ε=0.01.

The theoretical boundary of the cumulative reward is shown by the black dotted line in Figure 5, and it is calculated as an ideal condition, wherein the true parameter values are known and used for action selection from the second iteration. The cumulative reward for ε=0.01 with prior knowledge shows almost the same value as that in this theoretical bound. In other words, when prior knowledge is introduced, exploration with the policy $\pi_{random}$ is unnecessary, and the best performance can be attained by full exploitation with the policy π that maximizes the predicted reward from the beginning. On the other hand, in the case without prior knowledge in Figure 5B, the cumulative reward decreases significantly when ε=0.01 and 0.2 are compared with the case with prior knowledge in Figure 5A. In particular, when ε=0.01, the decrease in the second iteration is remarkable.

**Figure 6.**
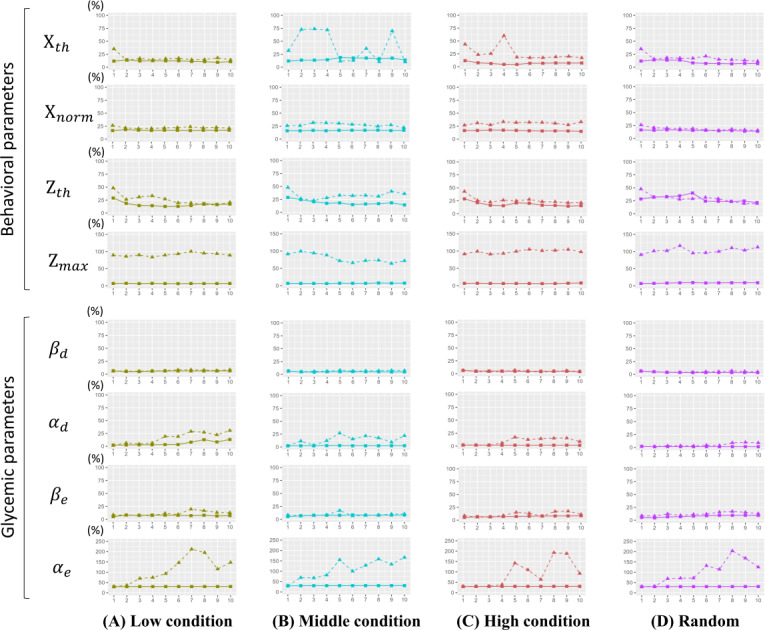
Average estimation error of behavioral parameters and glycemic parameters across different ε-greedy conditions: (A) low condition, (B) middle condition, (C) high condition, and (D) random. ■ represents results with prior, and ▲ represents results without prior. The vertical axis of each graph represents the average estimation error ($\epsilon_{p}\left(i\right)$) of the pth parameter among all 10 users, and the horizontal axis represents the iteration time (i) of the experiential learning.

Next, regarding question Q2, we show the actual changes in the parameter estimation error ($\epsilon_{p}\left(i\right)$) of the behavioral adherence and glycemic response models in Figure 6. First, it can be seen that the case without prior knowledge (▲) has a larger estimation error than the case with prior knowledge (■) for the first iteration. This large error leads to a prediction error of the reward for each action candidate and therefore makes it easier to select worse actions with the policy $\pi^{*}$. This may be the reason why the cumulative reward decreases immediately in case ε=0.01 and 0.2 due to the erroneous action selection at i=2 in Figure 5B. Furthermore, in the case without prior knowledge, it can be seen that the estimation errors of some parameters (such as $Z_{max},\alpha_{e}$) are not improved as iteration time of experiential learning cycle increases. This indicates the potential risk in real experiments, showing that it is impossible to collect the necessary training data for stable parameter learning, even when imposing heavy burdens of repeated interventions on users. However, when introducing prior knowledge, we can confirm that the parameter errors are minimized from the first iteration for most parameters. This suggests that introducing prior knowledge can eliminate the burden on users and maximize their cumulative rewards.

### Real-World Experiment Results

Based on the simulation results, we conducted a real-world experiment with a simplified algorithm involving a single update of the policy. According to the setting derived in the simulation experiment, we introduced prior knowledge for parameter learning, and we took the greedy policy with ε=0 for action selection.

**Figure 7.**
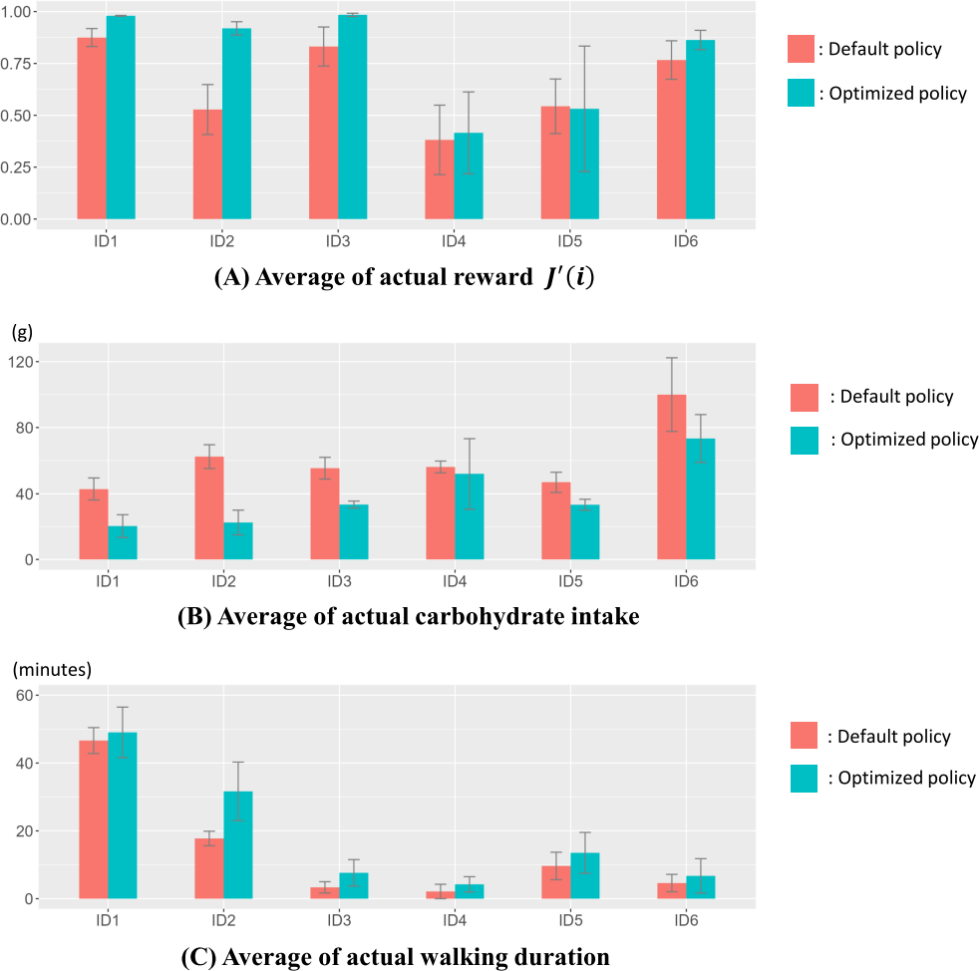
Changes in average reward (A), average carbohydrate intake (B), and average postprandial walking duration (C) for each participant in real experiments. Standard errors are shown as error bars. Statistically significant improvements (*P*<.05) were observed in each of reward, carbohydrate intake, and postprandial walking duration from the first experiment (default policy) to the second experiment (optimized policy). On average, reward increased from 0.65 (default policy) to 0.80 (optimized policy), carbohydrate intake decreased from 60.5 g (default policy) to 39.1 g (optimized policy), and postprandial walking duration increased from 14 minutes (default policy) to 18.8 minutes (optimized policy).

The obtained metric values for each participant from the real experiments conducted on the within-participant design are shown in Figure 7A. For all participants except ID5, the simple reward average increased in the second experiment using the proposed method. On average, for all participants, this metric increased from 0.65 in the first experiment to 0.80 in the second experiment, representing a 23% increase in reward. To statistically evaluate this improvement, we performed a paired *t* test comparing the average rewards between the first (default policy, R1) and second (optimized policy, R2) experiments across the 6 participants. The analysis revealed a statistically significant increase in reward following the optimized policy (*P*=.04). This result indicates that action selection using the proposed method is effective for real-world applications, consistent with the simulation results.

Moreover, we show how carbohydrate intake and postprandial walking duration changed in the first and second experiments in Figures 7B and 7C. All participants showed decreased carbohydrate intake and increased postprandial walking duration. For all participants, the average carbohydrate intake decreased by 35.3% from 60.5 to 39.1 g, and postprandial walking duration increased by 34.3% from 14.0 to 18.8 minutes. To further quantify these behavioral improvements, we conducted paired *t* tests for carbohydrate intake and postprandial walking duration. For carbohydrate intake, the mean difference (R2 – R1) was −21.425 g, and the paired *t* test showed a statistically significant reduction (*P*=.003). Similarly, for postprandial walking duration, the mean difference (R2 – R1) was 4.780 minutes, and the paired *t* test indicated a statistically significant increase (*P*=.03). These statistically significant behavioral changes underscore the effectiveness of the proposed method in guiding users toward healthier actions.

**Figure 8.**
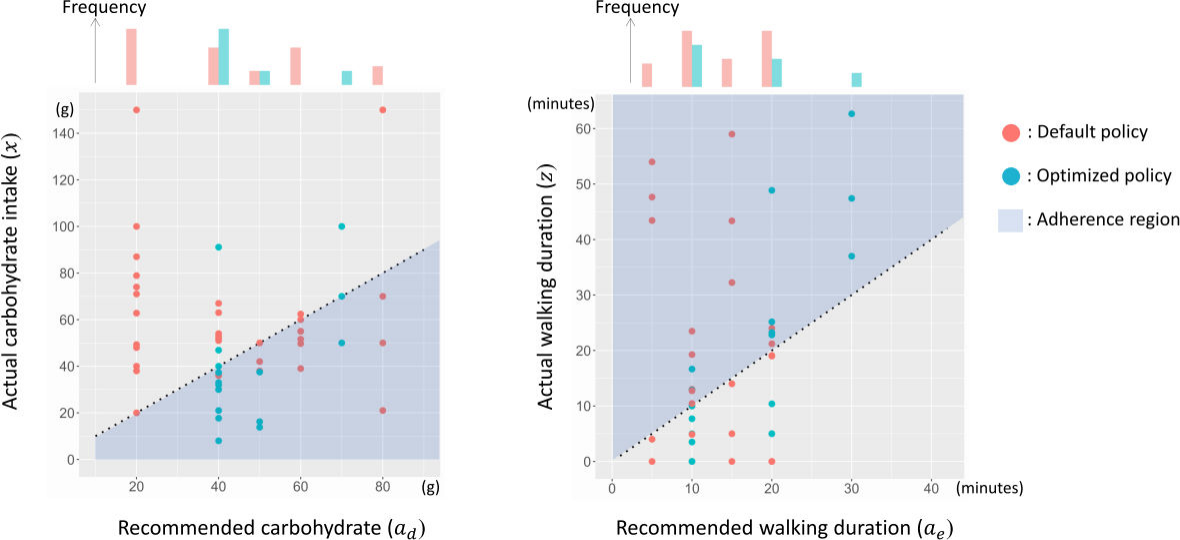
Recommended action vs actual behaviors in real experiments (left: carbohydrate intake and right: postprandial walking). The horizontal and vertical axes show the values of recommended action ($a_{d},a_{e}$) and actual behavior (x,z), respectively. Each data point denotes a pair of one action and one behavior for any participant. A participant is considered to adhere to the recommended action in cases $x\leq a_{d}$or $z\geq a_{e}$ shown in the blue region. For each policy, the frequency of action value is shown on top of each graph. The distribution differs between policies.

Furthermore, to investigate the impact on behavioral adherence, we show the actual behaviors of all participants in response to the recommended actions in Figure 8. The adherence to carbohydrate intake improved from the first experiment to the second experiment. In fact, as a result of calculating the adherence rate for each experiment, the adherence rate for carbohydrate intake increased from 45.7% to 83.3% and that for postprandial walking duration increased from 40% to 55.6%. These behavioral improvements support the hypothesis that action selection using the proposed method is appropriate for participants from the viewpoint of ease of behavior change.

## Discussion

### Principal Findings

In this paper, we propose a multiarmed bandit algorithm based on the two-stage reward prediction mechanism, which can individually plan mobile behavioral interventions and make it easier for users to perform actions and reduce glucose levels after eating.

From the simulation experiment results, first, we confirm that a larger cumulative reward can be obtained by adopting the proposed method than by selecting the default action. This demonstrates the effectiveness of our online algorithm for personalizing recommendations. Second, it is also demonstrated that the parameter learning converged immediately at the first iteration (i=1) by introducing prior knowledge. This indicates it is not always necessary to repeat the intervention experiment many times for obtaining accurate parameters, emphasizing the importance of the initial single update we conducted in the real-world feasibility study.

From the short-term real-world feasibility study, with a simplified proposed method involving a single update of the policy into a personalized one, it is found that actual postprandial glucose levels can be improved using the proposed method through behavioral improvements in both carbohydrate intake and postprandial walking. Specifically, we observed an average 23% improvement in actual glucose responses, which was statistically significant (*P*=.04), along with statistically significant improvements in behavioral adherence to the recommendations concerning carbohydrate intake (*P*=.003) and postprandial walking (*P*=.03). The adherence rates also increased notably (from 45.7% to 83.3% for carbohydrate intake and 40% to 55.6% for postprandial walking). While promising, these results are based on a small-scale pilot study with healthy participants and a simplified intervention protocol. Therefore, they should be viewed as preliminary evidence rather than definitive conclusions, primarily serving as guidance for the design of future, more comprehensive clinical trials. This demonstrates the potential effectiveness of the proposed method in a real-world setting. Crucially, this pilot demonstrated that even a single update, informed by our novel two-stage reward prediction model and based on initial user-specific data, can yield statistically significant improvements in clinical outcomes (glucose levels) and behavioral adherence in a real-world setting. This offers compelling initial evidence for the practical viability and immediate impact of our personalized approach, serving as a vital proof-of-concept for its core mechanism in a complex human context.

While simulation experiments elucidated the algorithm’s behavior across diverse iteration numbers (i), the real-world feasibility study, designed as a pilot, adopted a simplified approach: a single policy update (from default to optimized after the initial experiment), prioritizing feasibility and logistical tractability. This design inherently meant that the full extent of continuous learning and adaptive capabilities, central to multiarmed bandit frameworks over extended iterations, was not fully explored. Consequently, this study primarily demonstrated the potential of personalized recommendations derived from an initial learning phase, rather than evaluating continuous adaptive optimization over a prolonged period. This outcome underscores the critical need for future investigations to deploy the full online algorithm, addressing the challenges and benefits of continuous policy updates, particularly concerning user engagement and dynamic model accuracy.

To the best of our knowledge, this is the first attempt to (1) clarify how to optimize behavioral interventions for improving glucose levels and (2) demonstrate the effectiveness of the method through simulation experiments and real interventional experiments.

### Limitation

While this preliminary study offers promising insights, it is important to acknowledge several limitations that warrant future investigation to ensure comprehensive validation and generalization. First, the real experiment was short-term and small-scale. The findings from this preliminary study, which involved only 6 healthy participants over a short duration, should therefore be interpreted with caution and are not readily generalizable to a broader population, particularly including patients with diabetes who represent the primary target group for this intervention. The exclusion of patients with diabetes in this initial feasibility study naturally limits the immediate clinical applicability of these specific real-world results. While this real-world feasibility study offers encouraging initial evidence that effective personalized policy updates could be established within a practical, short-term data collection period (eg, 6 days), future research will include a long-term and massive intervention study. This is needed to fully evaluate the sustained effects and long-term stability of parameter convergence and policy updates beyond the initial phase (ie, after the third iteration [i≥3]) in diverse real-world settings and patient populations. Also, as our target in this study was limited to healthy participants, the interventional experiments in patients with diabetes should be performed for a comprehensive evaluation, adhering to robust statistical principles, including multiple comparison adjustments.

Second, this study primarily compared the proposed online algorithm against a randomized (default) policy. While this comparison effectively demonstrated the benefit of personalization over a nonpersonalized baseline, it did not include a direct comparison with other existing reinforcement learning-based or contextual bandit algorithms from the literature. Such comparisons would provide a more comprehensive understanding of the relative performance advantages and disadvantages of our proposed method against alternative state-of-the-art approaches. Future work could aim to include these comparisons, potentially through simulation studies based on established benchmarks or by incorporating other algorithms into real-world trials. Furthermore, a limitation exists in the parameterization of our simulation experiments. While parameter ranges for virtual users (Table 1) were based on literature, specific values were partially informed by our small-scale feasibility study (Table 2). This limited sample of 6 healthy participants may not be fully representative, potentially affecting the generalizability of simulation results. Consequently, these simulations primarily served to qualitatively explore the algorithm’s behavior and identify optimal operational settings (eg, epsilon values and importance of prior knowledge) for future large-scale trials, acknowledging they do not provide definitive quantitative predictions. Future research should prioritize parameter validation and derivation from larger, more diverse datasets, possibly through crowdsourcing or extensive clinical trials, to enhance simulation robustness.

Third, the predictive models, key to our two-stage reward prediction mechanism, possess inherent limitations, particularly regarding data requirements and accuracy. Furthermore, the two-stage prediction itself introduces a compounding effect of prediction errors: inaccuracies in behavioral adherence predictions can propagate and amplify errors in subsequent glycemic response prediction. This “multiplication” of noise impacts reward estimation, especially with limited data. While our preliminary study showed improvements in both glucose and behavior, the potential impact of such compounded prediction errors on long-term reward estimation and intervention effectiveness needs to be thoroughly evaluated in future large-scale studies. Finally, our behavior adherence model is simple, and user responses are also affected by external contextual factors such as weather or daily schedules [14], and adherence levels may decrease with engagement time [42,43]. Therefore, future development should focus on robustly handling limited initial data, efficient data collection strategies, precise quantification or mitigation of propagated uncertainty, and incorporating psychological and contextual factors into sophisticated behavioral models. Crucially, improving behavioral adherence through this future development is also key to minimizing the compounding effects of prediction errors within our two-stage model.

Fourth, the behavioral parameters’ prior knowledge, derived from self-reported questionnaires (Table 2), may not perfectly reflect true user tendencies due to cognitive biases. Inaccuracies in these initial priors could affect the model’s early parameter estimation accuracy and the speed of convergence to optimal recommendations. While our multiarmed bandit naturally adapts over time through experiential learning, more robust methods for acquiring these priors, such as integrating objective behavioral data, could further enhance initial performance and reduce the burden of exploration.

Finally, while our current action candidates are tailored around carbohydrate intake and postprandial walking, expanding the scope to include other types of exercise or dietary components would enhance personalization. Also, redesigning the reward function to align more closely with clinical guidelines for diabetes would improve its clinical relevance. Incorporating advanced behavioral change techniques [44], such as gamification, could further boost user engagement and usability.

### Future Directions

Beyond the immediate limitations, our study highlights several important directions for future research and practical implementation. Foremost among these, addressing the ethical considerations and practical challenges associated with long-term behavior modification interventions is crucial for successful clinical deployment. Factors such as user fatigue from continuous data collection and potential disengagement with recommendations over extended periods need careful consideration. Moreover, the current framework does not explicitly incorporate mechanisms to prevent the recommendation of potentially clinically damaging actions. While action candidates are prepared within a clinically plausible range, individual-specific contraindications or extreme physiological states could render even a seemingly “plausible” recommendation detrimental (eg, recommending a very low carbohydrate intake to someone with specific metabolic conditions). Future work will also include empirically establishing practical timelines for robust parameter convergence and policy updates, considering user engagement and burden.

Another critical area concerns the data requirements and accuracy for personalized recommendations. A significant challenge, especially for initial deployment, is the need for sufficient user-specific data to effectively learn and personalize recommendations. While our method uses prior knowledge to accelerate initial learning, acquiring this initial information (eg, via questionnaires or pretraining data) can be difficult and prone to cognitive biases, as self-reported behavioral priors (Table 2) may not perfectly reflect true user tendencies. However, recent advancements in mobile health apps, such as eMOM (Helsinki University Hospital) [8], are enabling the comprehensive collection and visualization of vast amounts of user behavioral and physiological data, including glucose levels. This digital self-tracking, by visualizing relevant data, has shown a positive impact on user understanding of their own health characteristics and offers a promising avenue for acquiring more robust and objective priors. For future clinical real-world applications, directly providing recommendations without proper context carries medical risks. Therefore, a clinician-in-the-loop approach is crucial. This involves an initial phase focused on data collection and visualization (eg, through mobile apps such as eMOM [8]), followed by a collaborative process where clinicians work with users to understand individual characteristics based on the visualized data. This systematic approach ensures the quantity and quality of prior information for each user before recommendations are made. Future work will integrate such an eMOM-like mobile app within a clinician-in-the-loop framework, enabling large-scale validation of the entire process from data visualization to personalized recommendation.

To achieve full clinical deployment, it is paramount to integrate safety constraints into the action selection process, developing methods to filter out adverse clinical outcomes (eg, through real-time physiological data integration or clinician-in-the-loop systems). Future work should proactively investigate strategies to mitigate these issues, such as dynamic adjustment of intervention intensity, gamification elements to maintain sustained engagement, and robust ethical frameworks ensuring user well-being, privacy, and safety in longitudinal deployments.

### Conclusions

In this study, we proposed a method for optimizing the planning of dietary and exercise recommendations to improve postprandial glucose levels through behavioral changes. The proposed method is a multiarmed bandit based on a two-stage reward prediction model, where an action is a combination of the amount of carbohydrate intake and postprandial walking duration, and the reward is the postprandial glucose level. We specifically realized the reward prediction for each action by predicting the behavioral responses to an action and then predicting the postprandial glycemic response using the predicted responses. From the simulation experiment, it was demonstrated that action selection using the proposed method significantly increased the cumulative reward compared to the default action selection. Simultaneously, we found that the most beneficial setting of the proposed method is adopting a policy to maximize the predicted rewards from the beginning, together with robust parameter learning using prior distributions for the prediction models. Finally, based on this finding, an intervention experiment was conducted on 6 healthy participants, and it was shown that the application of the proposed method provided preliminary evidence that it could improve postprandial glucose levels along with the behaviors of carbohydrate intake and postprandial walking. This initial real-world investigation, while valuable for understanding the practical considerations and informing the design of larger trials, underscores the need for extensive long-term validation in diverse patient populations, ensuring user safety and promoting sustained engagement.

## Supplementary material

10.2196/70826 Multimedia Appendix 1 Additional material on the two-stage reward prediction model and the simulation experiment setup.

## Abbreviations

iAUC: incremental area under the curve
