# Supplementary material for: Personalized Glucose Management With AI: Pilot Study Using a Multiarmed Bandit Approach
Source: JMIR Form Res. 2026 Mar 19;10:e70826. doi: 10.2196/70826 (PMC13010317; doi:10.2196/70826)
Supplement: Multimedia Appendix 1 [file formative-v10-e70826-s001.docx]

## **Supplemental section A: Two-stage Reward Prediction Model**


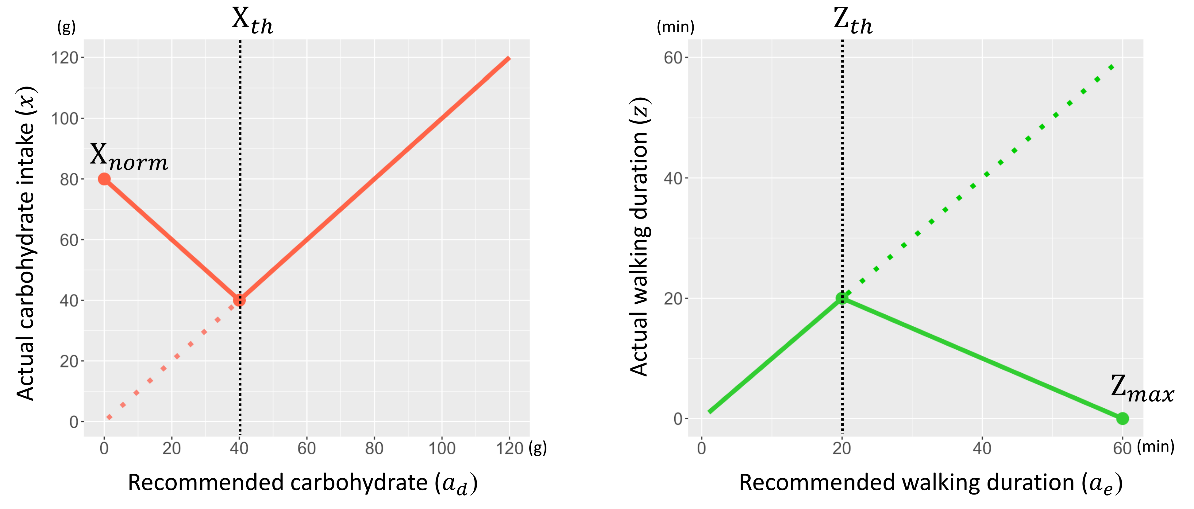


Figure S1: Dietary adherence model (left) and exercise adherence model (right)


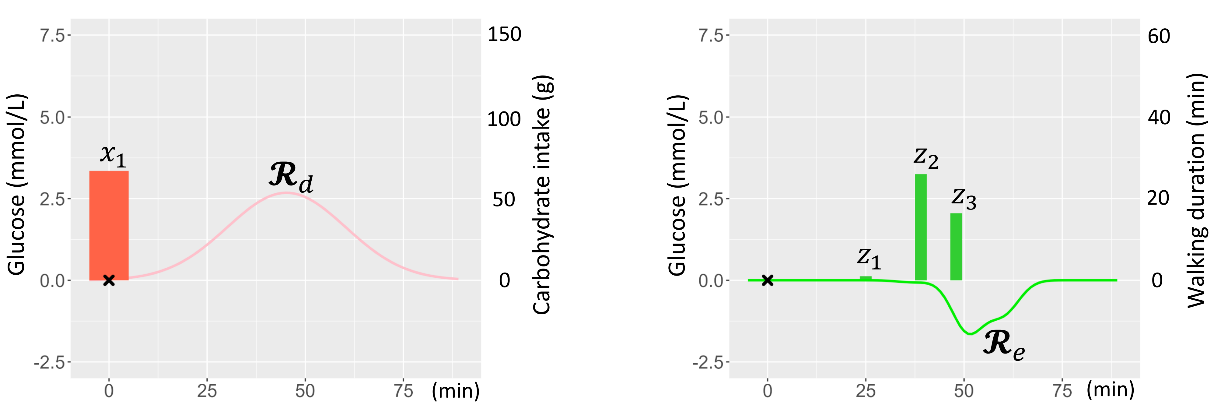


Figure S2: Dietary response curve (left) and exercise response curve (right) on glucose level

## **Supplemental section B: Simulation Experiment Setup**





Figure S3: User response simulator.


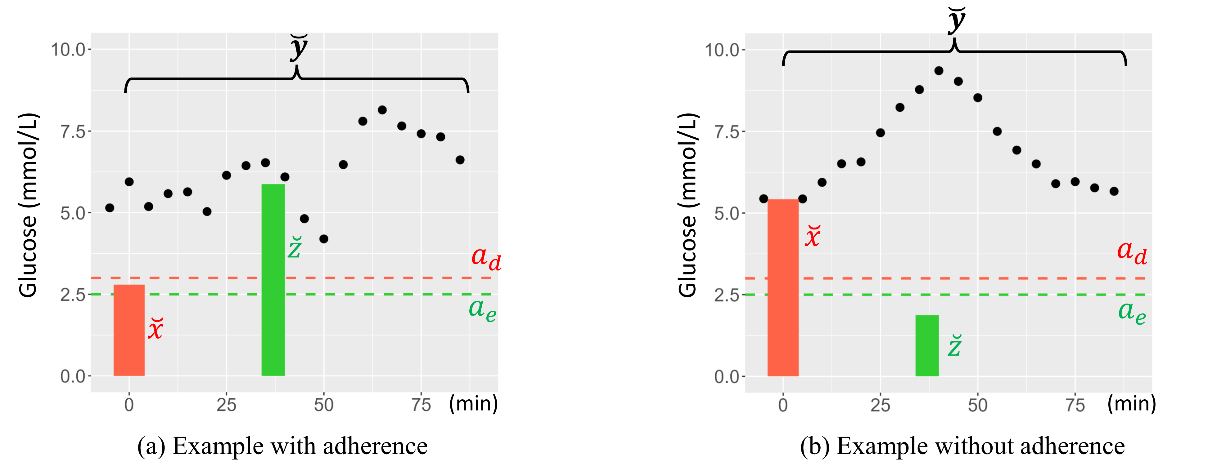


Figure S4: Examples of simulated responses of virtual user. In left example, user adhered to action ($a_{d},a_{e}$), took lower carbohydrate ($\breve{x}$), and walked for longer minutes ($\breve{z}$). Then, glucose levels didn’t increase much. In right example, user didn’t adhere to same action, causing significant increase in glucose levels.
